# Application of next-generation sequencing to identify different pathogens

**DOI:** 10.3389/fmicb.2023.1329330

**Published:** 2024-01-29

**Authors:** Aljuboori M. Nafea, Yuer Wang, Duanyang Wang, Ahmed M. Salama, Manal A. Aziz, Shan Xu, Yigang Tong

**Affiliations:** ^1^College of Life Science and Technology, Beijing University of Chemical Technology, Beijing, China; ^2^College of Medicine, Department of Microbiology, Ibn Sina University of Medical and Pharmaceutical Science, Baghdad, Iraq; ^3^State Key Laboratory of Chemical Resource Engineering, Beijing University of Chemical Technology, Beijing, China; ^4^Medical Laboratory at Sharkia Health Directorate, Ministry of Health, Sharkia, Egypt

**Keywords:** Sanger, next generation sequencing, pathogens, bacteria, fungi

## Abstract

Early and precise detection and identification of various pathogens are essential for epidemiological monitoring, disease management, and reducing the prevalence of clinical infectious diseases. Traditional pathogen detection techniques, which include mass spectrometry, biochemical tests, molecular testing, and culture-based methods, are limited in application and are time-consuming. Next generation sequencing (NGS) has emerged as an essential technology for identifying pathogens. NGS is a cutting-edge sequencing method with high throughput that can create massive volumes of sequences with a broad application prospects in the field of pathogen identification and diagnosis. In this review, we introduce NGS technology in detail, summarizes the application of NGS in that identification of different pathogens, including bacteria, fungi, and viruses, and analyze the challenges and outlook for using NGS to identify clinical pathogens. Thus, this work provides a theoretical basis for NGS studies and provides evidence to support the application of NGS in distinguishing various clinical pathogens.

## Introduction

For patients to be treated effectively, identification of the microorganisms that cause the infection is essential. The traditional pathogen identification methods include culture method, serological detection, and molecular biology methods (such as nucleic acid amplification). Nevertheless, not all bacterial species can be effectively cultured in the diagnostic laboratory, and novel pathogens cannot be detected by nucleic acid amplification. Some unknown pathogens can swiftly spark hospital epidemics that put patients in danger while they are being treated. DNA sequencing has made great advances in various disciplines, and specific progress has been made in personalized therapies (Jiang et al., 2022). The process of DNA sequencing can be used to determine the exact order of the nucleotide bases (adenine, cytosine, guanine, and thymine). Since the emergence of Frederick Sanger’s pioneering work in the 1970s, when he used the “plus and minus method” to sequence the first complete genome, DNA sequencing technology has been progressing quickly. Eventually, the Sanger chain termination or dideoxy technique, which was first described in 1977, laid the groundwork for the swift advancement of DNA sequencing technologies and made it possible to sequence the human genome for the first time in 2001 (Sanger and Coulson Nicklen, 1977). Sanger sequencing continues to be a popular technique, particularly for examining a small number of DNA sequences, as it offers high-quality DNA sequencing information for areas up to 1,000 bases. Sanger sequencing laid the foundation for the development of next-generation sequencing (NGS) technology.

The need for large-scale sequencing has quickly led to the development of NGS. Pyrosequencing, reversible-dye terminator, and proton detection are examples of the various NGS systems and methods that are created over time and based on unique chemistries and detection techniques (Garrido-Cardenas et al., 2017). NGS can be used to detect several infections. In contrast to conventional sequencing techniques, which allowed for the sequencing of one or a few very short DNA fragments that had previously been amplified by polymerase chain reaction (PCR), this cutting-edge technology has been a true revolution. NGS applications are increasingly prevalent, and they have evolved from study tools to diagnostic techniques. This review first presents detailed descriptions of Sanger and second-generation sequencing, which are the most used sequencing methods, in addition to a brief overview of the rapidly developing third-generation sequencing. Then, the application of NGS, including whole-genome sequencing (WGS), targeted next-generation sequencing (tNGS), and metagenomic next-generation sequencing (mNGS) (Mitchell and Simner, 2019), in clinical disease diagnosis is introduced. In addition, we focus on the clinical application of NGS for the identification of different pathogens, such as bacteria, fungi, and viruses. Finally, we discuss the challenges and the outlook of NGS in pathogen identification.

### Development of sequencing technologies

Sequencing technologies are categorized as first-generation sequencing (for example, Sanger), second-generation sequencing (for example, NGS), and third-generation sequencing (for example, nanopore sequencing).

The first generation consists of the Sanger and Maxam Gilbert procedures, which are two distinct techniques. Based on the chain-termination method, Sanger sequencing is more widely used. The developing chain in the chain termination method ends when dideoxynucleotides (ddNTPs) are incorporated. After being run on conventional slab gels, fragments of DNA varying in length (by a single nucleotide) were recovered, and the pattern of bands was used to determine the sequence. Fluorescently labeled ddNTPs (an automated sequencing approach) are used in place of radiolabeling, and the sequence is determined by varying the wavelength of the laser light (Smith et al., 1986). A maximum read length of 800–1,000 bp can be produced with this technique. The length of the sequenced fragment is the result of one run as using this technology only one fragment in a single capillary can be sequenced (Gupta and Verma, 2019).

The second-generation sequencing allows millions of sequencing reactions to occur simultaneously on a single solid surface, such as a glass slide or beads. This approach only requires the reaction to be spatially isolated rather than physically separated in a different well, lane or tube. As a result, thousands of millions of distinct reactions take place at the same time, leading to a significant reduction in both the overall cost and manpower when compared to other conventional approaches. Many commercial NGS systems that are based on various technologies but generally follow a basic pattern or steps have been developed. The most significant benefit of NGS is the capacity to extract sequence information from individual DNA fragments in a library, which does not require large amounts of DNA/RNA. Moreover, NGS allows for *de novo* assembly that does not rely on references or amplification (Sohn and Nam, 2018). Therefore, NGS can be used to identify unknown pathogens. However, NGS reads are short and require different computational methods to analyze the data. The price of NGS in China ranges from several thousand RMB to tens of thousands of RMB. In the United States, NGS costs approximately $99 for non-invasive prenatal testing and $2,500 for exome sequencing (Phillips et al., 2020). The use of NGS in clinical patients will become more common as the cost of NGS decreases and health insurance reimbursement increases. These techniques have significantly contributed to the study in several areas of life science and are being introduced in clinical laboratories more frequently, with several diagnostic uses in the fields of pathogen identification, oncology, and human genetics (Rhoads and Kin Fai, 2015; Yamada and Nomura, 2020).

Third-generation sequencing methods can be used to sequence individual DNA molecules in real time without the need for an amplification step. These methods have made sample preparation simpler and can provide single runs. Moreover, third-generation methods often yield larger reads, roughly a few kilobases in length, which addresses the challenges in read assembly. Moreover, significantly, this method can be used to identify different pathogens. The first nanopore sequencing instrument was called MinION, was licensed in 2007 by Oxford Nanopore Technologies, United Kingdom, and went on sale in May 2014. The core of this device contains a flow cell with 2048 individual nanopores that are organized into four groups, with 512 nanopores per group, and the nanopores are managed by an application-specific integrated circuit (ASIC). The following is a quick summary of the sequencing process: the adapters are ligated to either end of the fragments and adapters facilitate polymerase binding at the 5′ ends of the fragments and allow fragment capture. Furthermore, by concentrating the DNA fragments near the nanopore, these adapters increase the fragment capture rate one thousandfold. Additionally, by covalently binding the complementary strands to one another, these hairpin-like adapters enable the adjoined sequencing of two strands. The polymerase moves along the template strand upon fragment translocation via the nanopore, and the procedure is repeated for the complementary strand. As pieces pass through the nanopore, the sensor detects the shift in ionic charge. To ensure the corresponding duration, mean amplitude, and variation, the change in ionic charge or characteristic disruption in current is split into discrete occurrences. Ultimately, the sequence of events is deciphered by computer tools and graphical models (such as MinKNOW) to determine the nucleotide sequence. The data gathered from the complimentary and template strands are combined to provide the “2D read”(Jain et al., 2018). The potential of this method to advance pathogen detection and comprehension in many contexts is demonstrated by its capacity to identify specific viruses. For example, the effective handheld MinION sequencer enables the nontargeted detection of Ross River virus (RRV) using a metagenomic technique within a few hours (Batovska et al., 2017). The reads spanning up to 2.5 kb helped identify the virus, with an accuracy rate of more than 98%.

Here, we compare the first, second, and third generation sequencing (Table 1).

**Table 1:** Comparison of three sequencing technologies.

	First generation sequencing (Sanger method)	Second generation sequencing (NGS method)	Third generation sequencing (nanopore sequencing)	References
DNA amount	Usually requires a higher quantity of DNA (ranging from micrograms to milligrams), depending on the specific method and application.	Requires a comparatively little quantity of DNA, often ranging from nanograms to micrograms.	Without the need for an amplification step.	Gupta and Verma (2019), Deamer et al. (2016)
Quantification	While correct quantification is vital, the criteria are not as strict as for NGS.	Accurate measurement is essential because of the increased sensitivity and decreased input demands.	Quantitative PCR techniques were used at Harvard to quantify the number of DNA molecules that passed to the trans side of the pore.	Kiehn and Car (2017), Gupta and Verma (2019), Deamer et al. (2016)
Read Length	Produces extended read lengths, frequently reaching up to 1,000 bases or more.	Typically produces shorter read lengths than Sanger sequencing, although modern technologies have made some improvements.	Up to 2.273 megabases or more.	Nowrousian (2010), Gupta and Verma (2019), Wang et al. (2021)
Cost	Increased sequencing expenses.	Reduced expense per sequenced.	Higher costs, about $1,000.	Gupta and Verma (2019), Faulk (2023)
Speed	Lengthier procedure, particularly for large-scale projects.	Parallel processing capabilities result in faster turnaround times, facilitating rapid data creation.	Less time required, about a few hours.	Gupta and Verma (2019), Koch et al. (2023)
Throughput	The throughput is limited because to the sequential processing of individual DNA fragments.	Utilizing high throughput technology, this process allows for the simultaneous sequencing of millions of DNA fragments in parallel.	Increased to ~10–15 gigabases.	Slatko et al. (2018), Wang et al. (2021)

### Sanger method

**Figure 1:**
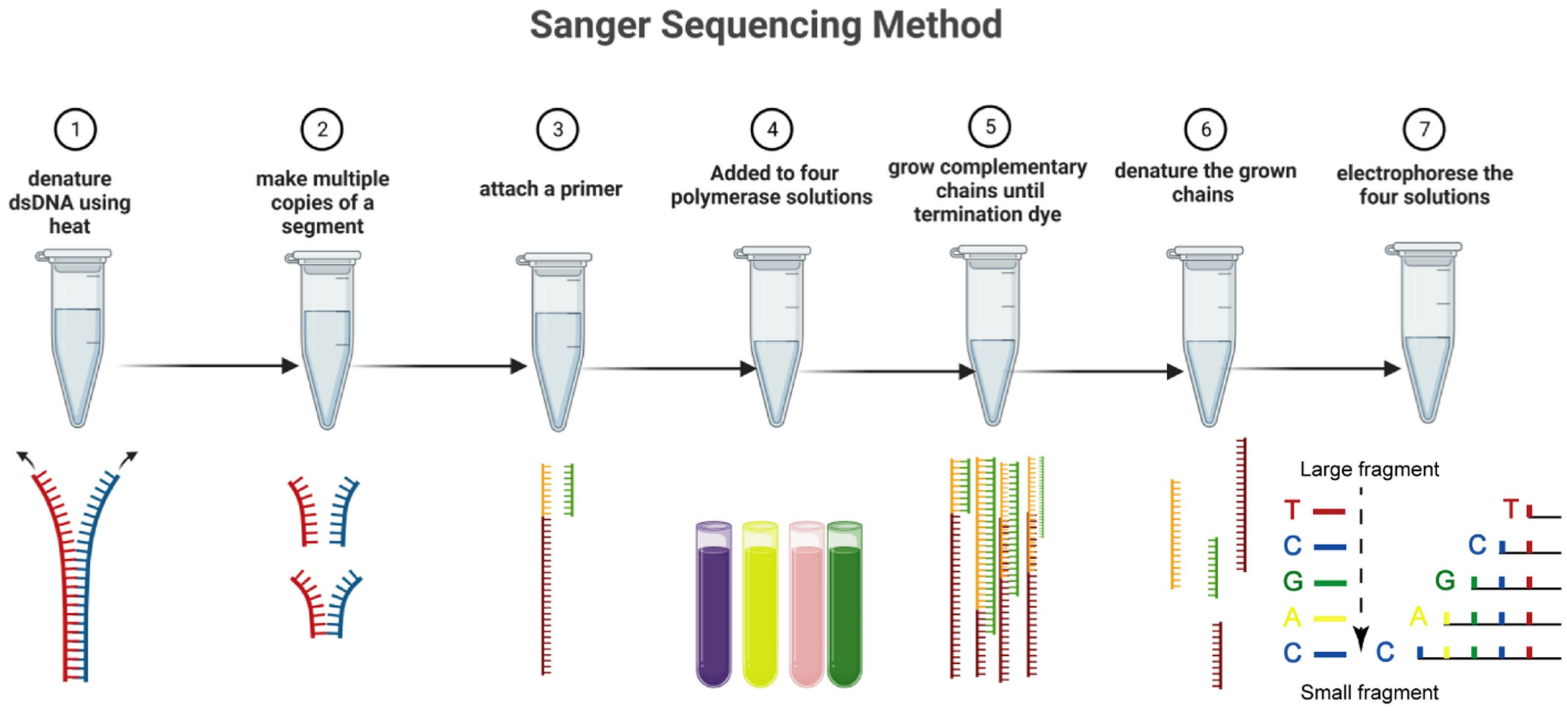
The Sanger sequencing method in seven steps. Including denaturing dsDNA, forming multiple copies of a segment, attaching primer, addition of polymerase solutions, amplifying the chains, denaturing chains, and electrophoreses solution. Dideoxynucleotides are labeled with four fluorescent markers, and the extension of the DNA strand is terminated when ddNTP is incorporated into the DNA strand. The resulting mixture of DNA fragments of different lengths is separated in capillary electrophoresis, and the base type and accuracy are determined by detecting the fluorescence intensity of different colors after laser irradiation.

The Sanger sequencing method has progressed via automation and commercialization. Although it has a slower sequencing speed than the NGS method, the Sanger method continues to be the best sequencing technique for many applications. The discovery of fluorescent dyes, the use of thermal cycle sequencing, which requires less input DNA, and the creation of thermostable polymerases to accurately and effectively insert terminator colors into the developing DNA strands are the three most important developments in Sanger sequencing (Figure 1) (Crossley et al., 2020). Specific nucleotides with end chains (dideoxy nucleotides) are used in Sanger sequencing as they do not include a 3’-OH group. As a result, DNA polymerase cannot create a phosphodiester bond, which causes the developing DNA chain to stop at that location. The ddNTPs are fluorescently or radioactively tagged for detection in automated sequencing instruments (Benner et al., 2007).

The most common use of Sanger sequencing technology is single-reaction sequencing operations that employ a particular DNA primer to a given template, such as those used to validate plasmid constructs or polymerase chain reaction products (Crossley et al., 2020). The popular Sanger sequencing technique makes use of time-consuming and low-cost molecular biological products, such as DNA purification reagents and kits, as well as inexpensive, high-quality manufactured primers (Sander et al., 1976). In addition, this technique can also be applied to determine the function of specific enzymes on fluorescently labeled DNA substrates by examining DNA fragment size. Various fluorescent labels, substrates, products, and reaction intermediates can be assessed using capillary electrophoresis in a single experiment. For instance, the kinetics of DNA polymerase and DNA ligase and several coupled enzyme processes, such as the processing of Okazaki fragments and ribonucleotide excision repair, can be detected by these methods. Moreover, researchers have performed high-throughput experiments using capillary electrophoresis (Greenough et al., 2015).

This technique is the most sophisticated for sequencing isolated genes and short tandem repeats. researchers have determined the source of many illness-related genetic mutations with the help of Sanger sequencing. However, the major drawback of this technique is that it is time-consuming because of the low conductivity. This technique only recognizes single-stranded mutations and can process short DNA sequences (300-1,000 base pairs) simultaneously. As a result, the demand for new technology to enable faster throughput sequencing of larger genomes at cheaper costs led to the creation of NGS technologies by using a wide range of inventive approaches (Rehm, 2013).

### Next-generation sequencing technology

NGS is distinguished by its fast and high-throughput properties. NGS can be used to obtain general data by deciphering millions of different DNA sequences at the same time (Petersen et al., 2019), making it possible to qualitatively investigate multiple types of genetic alterations (Manuscript and Malignancies, 2014). NGS is parallel sequencing, which can be used to simultaneously assesses multiple genes. NGS offers excellent throughput and speed and produces many sequences in a single run at a relatively low cost. NGS sequencing methods include the 454 Roche method, sequencing by oligonucleotideligation and detection (SOLiD), and Illumina. Here, we will focus on Illumina. The Illumina Solexa DNA sequencing system uses an eight-lane flow cell with oligonucleotide anchors to perform end repair, adenylation, and fragmentation of template DNA. Hybridization is facilitated by ligating adapters to the complementary anchors of the flow cell. “Bridge amplification” creates clusters, and sequencing is accomplished by incorporating fluorescently labeled reversible terminators (Adessi et al., 2000; Guo et al., 2008). Illumina’s methods, such as the MiSeq and HiSeq series, are industry leaders in NGS. The most recent models, the HiSeq 3,000 and HiSeq 4,000, use patterned flow cell technology and fall between the HiSeq X Ten and HiSeq 2,500 in terms of data output and run time (Reuterl et al., 2015). The smallest and most economical sequencer is the HiSeq 100.

Illumina’s sequencers are extensively utilized in the advancement of large-scale sequencing endeavors. The reasons for the widespread use of NGS technologies are multifaceted. First, these technologies offer exceptional accuracy in sequencing, ensuring reliable results. Additionally, the cost per gigabyte (Gb) of data obtained through these methods is quite low. Furthermore, the market offers a diverse range of equipment options, thereby allowing researchers to select the most suitable tools for their specific project requirements. The range of sequencing equipment varies from compact bench-top machines with moderate performance, such as MiniSeq, to large-scale instruments utilized for sequencing entire genomes in population-based initiatives, such as HiSeqX (Garrido-Cardenas et al., 2017).

Since NGS is capable of quickly identifying pathogens that threaten public health, it allows for health care workers to take urgent measures according to the type of pathogen to prevent or control large-scale spread. Therefore, NGS is of great significance in the clinical diagnosis of pathogens. For example, Köser et al. compared single nucleotide polymorphisms in clinical outbreak isolates of methicillin-resistant *Staphylococcus aureus* (*S. aureus*) using whole-genome sequencing, thereby providing health care professionals with rapid access to validated clinically relevant data (Köser et al., 2012). In addition, Greninger et al. detected the complete genome of *Balamuthia mandrillaris* in cerebrospinal fluid samples from patients with primary amoebic meningoencephalitis (PAM) using NGS, and the diagnosis was confirmed by the Centers for Disease Control and Prevention (CDC) using PCR (Greninger et al., 2015). NGS can also serve as a powerful tool for identifying virulence factors of pathogens, which can improve public health responses (Gilchrist et al., 2015; Li et al., 2016). In addition, NGS can play a role in infection prevention, with phylogenetic analysis of isolates facilitating the prevention of large-scale outbreaks of pathogens (Zhou et al., 2016).

Library construction for NGS has been developed and distributed in commercial kits, such as the NEBNext Ultra II Directional RNA Library Prep Kit (Illumina, San Diego, CA, United States), so that health care professionals can build the library according to the manufacturer’s instructions, which reduces the difficulty of the process. Taking RNA library building as an example, this process is simply divided into the steps of RNA fragmentation and priming, first-strand cDNA synthesis, second-strand cDNA synthesis, end prep of cDNA library, adaptor ligation, and PCR enrichment of adaptor-ligated DNA. The libraries are sequenced to obtain raw reads stored in fastaq file format, which contains the sequence information of the reads as well as the sequencing quality information. Not all reads are meaningful for analysis, and some short reads without overlap cannot be assembled into contigs. After removing low-quality and short reads, reads with overlapping regions are assembled into contigs and compared to known sequences by blastn and blastx. Subsequently, the complete sequence can be obtained through PCR detection and Sanger sequencing. The obtained sequences are aligned with known sequences, and the maximal likelihood tree or neighbor-joining tree is constructed to determine which branch it clusters with (Podnar et al., 2014; Tian et al., 2023).

**Figure 2:**
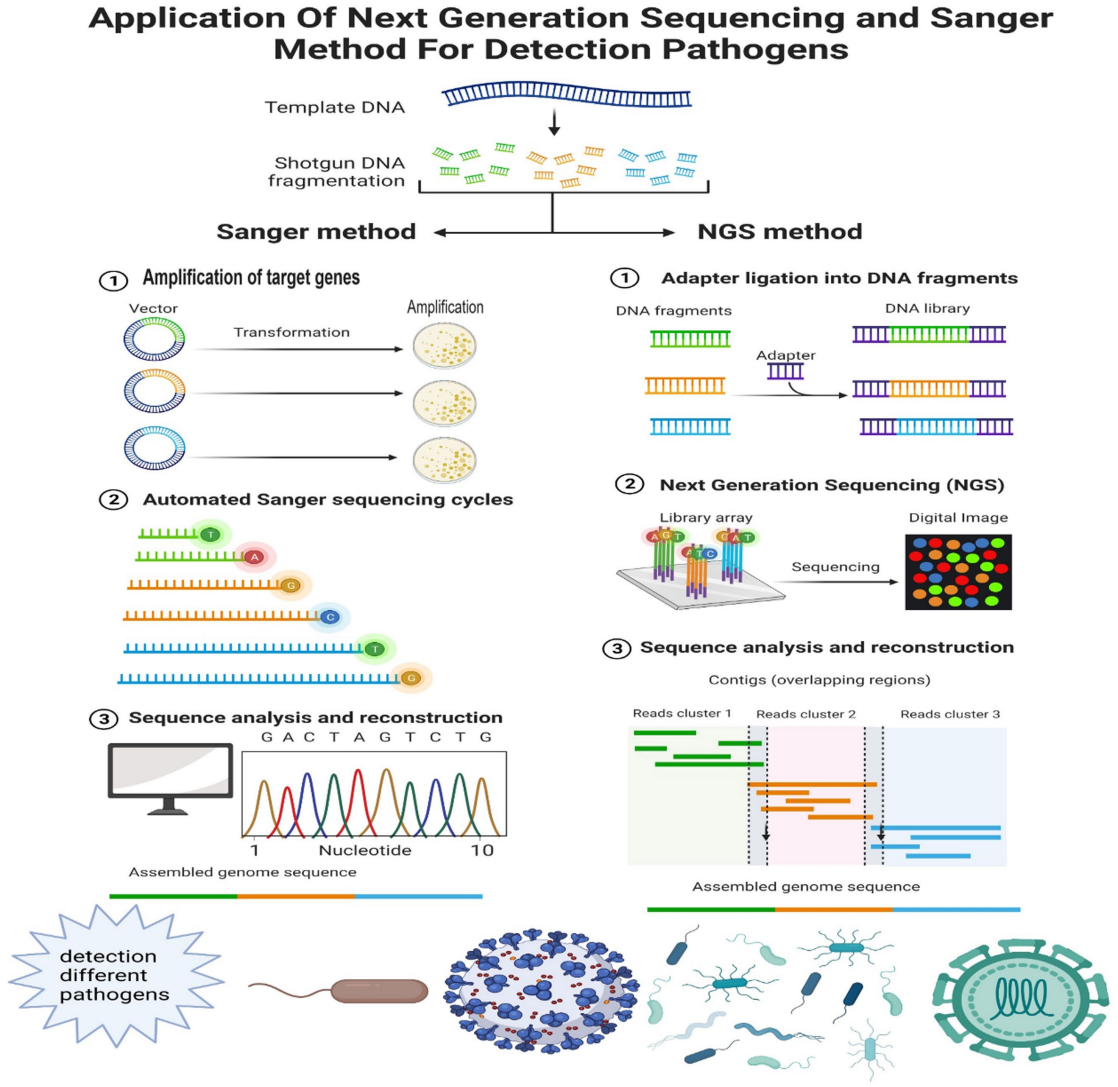
Comparing the different processes of the Sanger method and NGS in detecting different pathogens. Sanger method’s methodology comprises amplification, automated cycling, sequence analysis, and reconstruction. This technique generates several copies of the target DNA region. The workflow of NGS (take Illumina for example) in clinical setup, including sample separation and preparation, NGS based on the addressed request, Illumina process, related bioinformatics data processing, and retrieval of the final results. The different procedures of two methods result in different application in detecting various pathogens, including viruses, bacteria, and fungi.

Sometimes, only a few reads from pathogen signature sequences are sufficient to identify the pathogen. For example, bacterial species can be identified based on the analysis of 16S rRNA-related reads (Church et al., 2020). 16S rRNA-based analysis facilitates the study of complex microbial communities (Tourlousse et al., 2017; Dai et al., 2022). Jin et al. used single-base accurate cellular barcoded 16S rRNA sequences to identify individual bacteria to study the microbiota (Jin et al., 2022). In addition, Mukherjee et al. showed that 16-23S-based intergenic spacer regions (ISRs) improved the accuracy of bacterial community analysis at the subspecies level compared to 16S rRNA (Mukherjee et al., 2018). Analysis of the sequence of the 18S rRNA gene based on related reads can be used to identify fungi (Petri et al., 2019). For example, Zahedi et al. identified multiple fungi from 49 eukaryotic phyla in wastewater based on 18S rRNA NGS (Zahedi et al., 2019; Figure 2).

The core principle of most NGS technology is sequencing by synthesis (SBS). The DNA molecule to be sequenced is replicated (synthesized) by using DNA polymerases and dNTPs. To determine the base type of the incorporated nucleotide as the DNA molecule extends, nucleotides are either modified with distinguishing tags, such as fluorophores, or via the stimulated fluorescence of other substrates. In addition, NGS can also apply the principle of sequencing by hybridization (SBH). SBH is a method that assembles several overlapping oligonucleotide sequences to identify the DNA sequence of an organism. The foundation of SBH is the renaturing of complementary DNA strands following melting. As a consequence, the oligonucleotide probes hybridize in a way that makes it possible to identify the complementary sequences in the DNA target.

The advantages of NGS technologies promote the development of molecular biology by facilitating large-scale whole-genome sequencing (Pmg et al., 2019). NGS technology is usually used in multilevel studies for genomics, transcriptomics, and epigenomics. Other methods of pathogen identification include PCR-based and microarray assays. PCR-based assays can only be used to identify known pathogens. Microarray technology for pathogen detection differs in methodology depending on the specific pathogens being targeted, the design of the probes, and the method used for the array. The advantages of this approach include its affordability and multiplex analysis (Green and Pass, 2005). Limitations include insufficient probe density, data noise, and substantial initial expenses (Wang et al., 2002, 2003). NGS data is considered more quantifiable than microarray data (Radovich and Ragoussis, 2014; Gaston et al., 2022). Microarrays can be practical for recognizing DNA methylation (Han et al., 2019) and can also be used for non-model organisms. The microarray, however, is limited to classic organisms. Because of the large number of genes, efficient bioinformatics approaches need to be established to assess the significant amount of sequencing information gained in these experiment (Schmieder and Edwards, 2011). At the same time, not all reads are meaningful for analysis, and some short reads without overlap cannot be assembled into contigs (Hung and Weng, 2017). Here, we compare the different processes of the Sanger method and NGS in detecting different pathogens in Figure 2.

### Whole-genome sequencing

WGS is the assembly and sequencing of an organism’s whole genome and is currently the most frequently used technology to identify unknown organisms. In addition, NGS can be utilized to detect known microorganisms, and other approaches for detecting known microorganisms include mass spectrometry, culture, etc. (Bauermeister et al., 2022). The technological process of WGS differs depending on the type of organism. WGS of viral genomes is usually carried out directly from patient samples and does not call for the culture or isolation of the virus. In contrast, when attempting to detect infectious bacteria in a clinic, the presence of additional bacteria in clinical specimens that are either clinically insignificant, such as normal skin flora, or represent polymicrobial infections may complicate WGS results. Therefore, the bacteria must first be isolated and cultured to prepare for the extraction and sequencing of bacterial nucleic acids using WGS. Thus, WGS has evident limitations when the microorganism is impossible or difficult to cultivate. By monitoring outbreaks, WGS has proven particularly helpful in hospital and public health epidemiology research (Brown et al., 2015). For instance, WGS of clinical *Mycobacterium tuberculosis* (*M. tuberculosis*) has been performed to diagnose tuberculosis. In addition, WGS was able to identify and track the spread of *Klebsiella pneumoniae* isolates that produced CTX-M-15 or carbapenemase to resist colistin, thereby guiding infection control measures and halting the spread of these multidrug-resistant pathogens. Unlike Sanger sequencing of the hexon gene, WGS of adenovirus genomes isolated from patients in the neonatal intensive care unit can be used to identify the adenovirus species and genome characteristics to develop better therapeutic strategies (Zhou et al., 2015).

In addition to pathogen identification, WGS can offer information regarding pathogen virulence and novel resistance mechanisms. Identifying virulence factor genes, which are not detected by clinical laboratories or used in patient treatment, allows for the further study of virulence. One study, for instance, highlighted the potential use of WGS in identifying and classifying specific virulence genes in *S. aureus*, such as *S. aureus* toxins and panton valentine leucocidin (PVL) (Leopold et al., 2014). WGS can also be used for the early detection of novel resistance mechanisms that conventional molecular detection techniques may overlook, such as PCR of a particular gene or locus. The ability to identify resistant subpopulations is one of the biggest potential benefits of the viral WGS approach, which the Sanger method cannot achieve. According to one study, WGS of HIV improves the ability to find low-frequency drug-resistant mutations in HIV-1 (Tzou et al., 2018). Antimicrobial resistance (AMR) prediction is one of the most intriguing potential uses of WGS, and it may offer preliminary results more rapidly than conventional phenotypic methods. Furthermore, virulence factors and AMR genes can be detected by metagenomics (Sanabria et al., 2021).

With the correlation between antimicrobial resistance genes (ARGs) and phenotypic outcomes, numerous published reports have shown the potential of using WGS for identifying resistance in a range of bacteria. Nevertheless, comprehending the complex relationship between ARGs and phenotypic resistance is a complex task. Finding an ARG does not always imply that the gene is expressed; for example, regulatory processes and environmental circumstances might affect gene translation (Nielsen and Browne, 2022). Furthermore, using databases to identify ARGs is difficult. These databases might not always be complete or current, which could result in inadequate knowledge of the resistome. Genetic changes can confer resistance without the direct participation of recognized ARGs, which further complicates issues. This emphasizes the necessity of thorough genomic analysis that putative variations resulting from mutational events in addition to known resistance genes (Mao and Zhang, 2023). Extrachromosomal DNA fragments called plasmids are essential for the spread of ARGs. The link between genotypic and phenotypic resistance can become increasingly complicated when bacteria acquire plasmids that confer resistance. A more comprehensive understanding of the variables driving the spread of antibiotic resistance among bacterial populations can be obtained by examining plasmid-mediated resistance mechanisms (Dewan and Uecker, 2023).

In conclusion, improving our understanding of antimicrobial resistance will require determining the complex interactions between ARGs and phenotypic resistance. A multifaceted strategy that integrates genotypic and phenotypic investigations is necessary because of the constraints related to gene expression, mutations, plasmid-mediated resistance, database accuracy, and mutations. By combining sophisticated molecular approaches with strategies such As The Kirby-Bauer method, antimicrobial resistance dynamics can be explored in greater detail and open the door for focused interventions and efficient resistance management (Hughes and Andersson, 2017).

### Next-generation targeted sequencing

Before creating libraries and sequencing, in tNGS, known target sequences are amplified and enriched. The benefit of using tNGS over a metagenomic method is avoiding the needle-in-the-haystack problem of amplifying sparse microbial sequences in samples with a high proportion of host cells (Schlaberg et al., 2017). However, selection procedures, such as multiplex PCR for particular genes, could result in target bias. Although assays may also target genes associated with antibiotic resistance, the primary objective of tNGS in clinical applications is to identify microbial pathogens or pathogens in patient samples. PCR amplifies the 16S ribosomal RNA (rRNA) gene before NGS, which is the most popular enrichment technique for clinical applications and microbiome research (Salipante et al., 2014). Alternative enrichment and sequencing techniques are also being created. The development of a bacterial tNGS assay by amplifying and sequencing the complete 16S-23S rRNA region in urine or blood samples from patients with suspected urinary tract infections was reported by Sabat et al. (2017). Interpreting 16S data frequently only yields genus-level identifications, and the addition of 23S regions improves the specificity and sensitivity. In addition, 16S amplicon sequencing is also considered metagenomic. Although rRNA microarrays can also detect 16S rRNA, distinguishing between closely related strains is difficult with this data compared to NGS data (Zabarovsky et al., 2003; Pozhitkov et al., 2006). In addition, the range of species that can be detected using microarrays is limited, and novel or unknown organisms cannot be identified. tNGS was used to accurately discover the viral resistance of cytomegalovirus (CMV) in clinical samples, especially minor variants (Briese et al., 2015).

### Metagenomic next-generation sequencing

Compared to tNGS, which amplifies specific gene fragments, mNGS can be used to detect all genetic material. Therefore, in comparison to tNGS, mNGS has the advantage of detecting potential pathogens and has a wide detection range (Gaston et al., 2022; Gauthier et al., 2023). However, there are the disadvantages of unstable detection, higher cost and long detection time. All microbial groups (including bacterial, viral, and fungal agents), resistance markers, virulence factors, or even host biomarkers associated with various disease states can be sequenced simultaneously with unbiased detection. A straightforward diagnosis from patient samples can be made according to mNGS data. mNGS can detect cell-free DNA (cfDNA) or cell-free RNA (cfRNA) to identify pathogens. Importantly, whether the pathogens discovered based on mNGS are responsible for the disease needs to be carefully determined. Because these novel pathogens have been less researched in the past, they may be overlooked.

A few current studies indicate that mNGS has improved sensitivity when compared with current diagnostic methods, and these result indicate the potential of mNGS. A retrospective chart review revealed that the total clinical sensitivity and specificity for mNGS were 50.7 and 85.7%, respectively, while those for standard diagnostics were 35.2 and 89.1%, respectively (Mitchell and Simner, 2019). mNGS performs particularly well for fungi, viruses, anaerobes, and *M. tuberculosis*. For example, when the microorganism culture is negative, mNGS of sonicated fluids or synovial fluid from prosthetic joint infection gives an incremental 25 and 18.3% yield, respectively. One HIV-1, two Taenia solium, four fungi, and one other pathogen were found in the cerebral fluid of 94 patients who had subacute or chronic meningitis by using mNGS (Mitchell and Simner, 2019).

### Application of NGS for the detection of different pathogens

Infectious diseases are still a leading cause of human morbidity and mortality globally. The rapid and precise diagnosis of aetiologic microorganisms can promote the therapeutic process. The diversity of detectable microorganisms is relatively narrow when using culture methodology (Tomblyn et al., 2009; Miao et al., 2018), including inaccurate and time-consuming identification techniques involving pathogen isolation, selective culture, and pathological inspection. Clinical specimens may not yield conclusive results for cultivating pathogenic bacteria for days to weeks (Arastehfar et al., 2019).

Furthermore, the reported turnaround time for NGS requires substantially less time from receiving clinical samples to data analysis than traditional methods (Breitwieser et al., 2018). There are several reported practical uses of NGS in clinical settings (Ondov et al., 2011; Maabar et al., 2019). The results of NGS provide useful information for diagnoses, controlled treatment, the evaluation of efficiency, and the prognosis of infectious diseases (Murkey et al., 2017; Kufner et al., 2019). We summarize the applications of NGS in identifying bacteria, fungi, and viruses in detail to further promote its clinical use. Table 2 shows the advantages and disadvantages of NGS in the detection of different pathogens.

**Table 2:** Advantages and disadvantages of sequencing technology in the detection of different pathogens.

Pathogen	Advantage	Disadvantage	Sequencing approach	References
Bacteria	Simplified bioinformatics Amplifies the amount of microorganisms present and targets Reduced price	Only Bacteria identification Particular microbes Can still miss some species due to primer mismatches Scarce copy of pathogen sequences may be harder to detect.	tNGS (16S rRNA)	Petersen et al. (2019)
Fungi	Reduced price Better sensitivity and massive information More speed Simplified bioinformatics The ability of amplified most of the fungus is available.	Still miss some species due to primer mismatches Particular to a subgroup of parasites and fungus Scarce copy of pathogen sequences may be harder to detect	tNGS (18S rRNA gene/ITS gene sequence)	Xing et al. (2022), Arastehfar et al. (2019)
Virus	Higher sensitivity and amount of data allows *de novo* assembly and even reads level detect Potential to detect the full spectrum of viruses, including unknown and unexpected viruses Allowing for the thorough identification of minority variants, which represents a clear advantage over direct (Sanger) sequencing Detection of pathogens that do not rely on references or amplification	Assembly and characterization of complex/highly repetitive genomic regions Reconstruction of complete “real” viral haplotypes	WGS; mNGS	Barzon et al. (2011), Quer et al. (2022) Houldcroft et al. (2017)

### Bacteria

NGS shows high resolution regarding the bacterial genotypes and is a significant and powerful method in infectious disease epidemiology (Tweedy et al., 2018). The composition of causative microorganisms, such as gram-negative bacteria, gram-positive bacteria, anaerobes, and fungi, differs in septic patients in dissimilar clinical circumstances. In approximately half of sepsis patients, the causative organisms remain unidentified, specifically, culture-negative sepsis. Several investigations have revealed that genomic RNA or DNA fragments are linked with specific microorganisms. As a consequence, NGS of cfRNA or cfDNA in cleansed plasma can be applied to identify pathogens in sepsis samples, alongside information concerning genetic relatedness (Tassios and Moran-Gilad, 2018).

Globally, WGS may be beneficial to track the spread of bacterial pathogens, which have shown how quickly infectious diseases spread throughout the world (Harris et al., 2010; Jain et al., 2013; Katz et al., 2014). Using this technology, researchers observed a genomic change in a sensitive bacterial strain with resistance to the international disease Cholera (Mutreja et al., 2013). In addition, genomic mutations were observed in *Streptococcus pneumoniae* in a large group of specimens (Croucher et al., 2013). WGS can also help clarify the bacterial transmission between population groups by sampling bacteria from specific host groups. For instance, many studies investigate gonococcal genomic epidemiology (Grad et al., 2014). Transmission can occur between men who have sex with other individuals, which means that gonorrhea can be transmitted from one to another.

Additionally, WGS has shown advantages over alternative genotyping approaches for tracking and analyzing micro-epidemics; for example, WGS was used to compare 86 human *M. tuberculosis* isolates from a German outbreak (Roetzer et al., 2013; Bosch et al., 2016). WGS was employed in 2010 to analyze 63 methicillin-resistant *S. aureus* (MRSA) strains from diverse nations, and the results allowed for the reconstruction of intercontinental transmissions over a forty-year period, as well as the possibility for transmission within a hospital setting (Harris et al., 2010). The cholera outbreak in Haiti was also investigated using WGS, and it was discovered that the Haitian strains were closely related to those from Nepal (Jain et al., 2013; Katz et al., 2014). These groundbreaking experiments prove the utility of WGS for retroactive genotyping. Sequencing methods need to be improved to make WGS a plausible genotyping tool during epidemics (Fournier et al., 2014).

### Fungi

After years of focus on human-associated bacteria, human-associated fungi have progressively drawn attention. Clinical researchers have focused on fungal populations, especially Candida, Malassezia, Penicillium, etc., because of the generalization and rapidity of fungal diseases. The analysis and results of fungi detection can be influenced by the techniques utilized to analyze human fungal communities (Nilsson et al., 2019). The use of NGS in fungal diagnosis should be considered due to the lack of effective detection methods for clinical fungal infections and the seriousness of the fungal illness. NGS technology shows several advantages in detecting fungal pathogens. First, NGS technology is appropriate for microbial diseases caused by hostile cultures and slowly growing microbes, including fungi (Xing et al., 2022). In addition, NGS is a helpful tool for samples with low fungal loads (Sequencing et al., 2021). Second, NGS is considerably more specific than other approaches and offers more accurate identification of fungal species (Arastehfar et al., 2019). Finally, compared with first generation DNA sequencing, NGS exhibits better sensitivity and provides large amounts of information in addition to accuracy and speed.

However, the use of NGS to identify fungi has been relatively less researched during the last ten years. By the end of 2014, most bacterial and viral genomes had been sequenced with NGS, while many fungal genomes remained undiscovered (Thomma et al., 2016). Existing NGS technologies are only partially believable due to technical issues and objective mistakes (Nilsson et al., 2019). Leho Tedersoo’s group team focused on fungus and suggested that the existing NGS technique frequently conceals numerous significant and minor problems. Moreover, mycobiome sequencing requires both the repeatability of fungal sequencing data and the accessibility of public data. His research team also conducted a study assessing the impact of the Respiratory Pathogen ID/AMR (RPIP) kit on a specific NGS workflow. After thorough comparisons, they concluded that NGS workflows could not replace traditional culture and other methodologies, partially due to the complexity of bioinformatic analysis of NGS (Gaston et al., 2022). Additionally, various internal transcribed spacer (ITS) primer types could inadvertently result in the identification of several fungal species. For instance, although ITS1-F, ITS1, ITS5, etc., have a preference for basidiomycete amplification, ITS2, ITS3, ITS4, etc., have a preference for ascomycetes (Bellemain et al., 2010).

Further advancements should be made to improve the fungal genome database and next-generation detection techniques. Overall, the applications of NGS are expanding rapidly, from cutting-edge diagnostic techniques to common clinical detection. NGS may now be utilized for routine microbial detection because the speed of detection has increased. Genomic testing has become more popular, particularly since 2005, thanks to the development and advancement of NGS technology and the lower cost of the testing supplies (Fournier et al., 2014). The repeatability, quantification outcomes, and classification accuracy of NGS should be enhanced to differentiate a more comprehensive range of species. Consequently, there is still a long way to go before NGS can be a standard operating procedure for fungal detection in diagnostic laboratories.

### Viruses

The application of NGS for virus identification has become increasingly popular. In addition, NGS offers a cutting-edge tool for massive the large-scale genomic sequencing of viruses such as Hantaan virus (HTNV), hepatitis C virus (HCV), and coronavirus. NGS has opened a new era of viral genomics for the surveillance, tracing, and risk management of viral diseases. Here, we will concentrate on the viruses mentioned above.

Complete genome sequencing and the isolation of infectious particles are essential to define and develop preventive measures for HTNV epidemics. Dong Hyun Song et al. used the lung tissues of striped field mice to isolate 12 HTNVs in highly Hemorrhagic Fever With Renal Syndrome (HFRS)-endemic regions. To obtain the genomic sequence of HTNV isolates, sequence-independent, single-primer amplification (SISPA) NGS was used. Based on the entire length of the prototype HTNV 76–118, the nucleotide sequences of the HTNV S, M, and L segments were covered to 99.4–100%, 97.5–100, and 95.6–99.8%, respectively (Ganzenmueller et al., 2013).

In the new world of direct-acting antiviral (DAA) medicines, NGS technologies for HCV can detect both viral genotypes and genetic resistance. In one study, the ability of NGS techniques to identify full-length, deep HCV sequences and their usefulness for clinical diagnosis were assessed. They examined the following three NGS techniques used in four UK centers: (i) metagenomics, (ii) pre-enrichment of HCV RNA by probe capture, and (iii) HCV preamplification by PCR. A panel of samples with various viral loads and genotypes was used to compare the sequencing coverage, depth metrics, quasispecies diversity, detection of DAA resistance-associated variations (RAVs), mixed HCV genotypes, and other coinfections (Thomson et al., 2016). Nearly complete genome sequences were produced by each NGS technique from more than 90% of the samples. For samples with low virus loads, enrichment techniques and PCR preamplification provided better sequencing depth and efficiency. All NGS techniques correctly identified the mix of HCV genotypes in infections. Most RAVs were reliably discovered, and consensus sequences produced by various NGS techniques were generally consistent. However, the ability of various techniques to find identify RAV minor is varied. Human pestivirus coinfections have been discovered using metagenomic techniques.

In addition, NGS has also played a tremendously important role in the identification of severe acute respiratory syndrome coronavirus 2 (SARS-CoV-2) variants and the traceability of SARS-CoV-2 (Chan et al., 2020). For example, Pan et al. used NGS to obtain 2,994 whole-genome sequences of SARS-CoV-2 and performed phylogenetic and population dynamics analyses to rapidly identify the variants and lineages (Pan et al., 2023). The broad use of NGS in diagnosis would significantly improve the capacity of researchers and governments to keep track of new strains of infectious diseases such as SARS-CoV-2. Massive efforts are made to coordinate extensive sequencing of the SARS-CoV-2 virus, as well as identifying SARS-CoV-2 and coinfections using amplicon and metagenomic-MinilON-based sequencing, respectively. By using primers targeting highly conserved regions of a genome, amplicon-based NGS technology is frequently used to offer detailed information of targeted region. mNGS, in contrast, employs a shotgun strategy to identify all genetic material in a sample, as opposed to the highly conserved genetic region.

Researchers are looking to improve NGS technology in SARS-CoV-2 detection. The amplicon-based sequencing method provided a significantly higher read depth than the metagenomic approach. For instance, SARS-CoV-2 was discovered in nasopharyngeal swab material from a patient in Feira de Santana-Bahia, Brazil, using meta-transcriptomic NGS (Campos et al., 2020). To eliminate rRNA from one sample, they used Thermo Fisher’s Low Input RiboMinus TM Eukaryote System v2 for the ion-semiconductor sequencing utilized in this procedure. Human transcripts comprised 77.29% of all reads in the rRNA-depleted sample, while 84.49% of all reads in the whole RNA library were human transcripts. Despite the inefficiency of host genome removal, the genome coverage of contigs in the rRNA-depleted library increased 30% compared to nondepleted samples. How to effectively remove host contamination is also one of the problems that needs to be solved (Song and Xie, 2020). These results imply that rRNA depletion methods may improve the diagnostic capabilities of NGS (Liu et al., 2020).

## Conclusion and prospects

Outcomes following NGS application for diagnosing infectious illnesses are heartening. The use of NGS in pathogen identification has several benefits over conventional approaches, including the capacity to study numerous pathogens at once, high sensitivity and specificity, and the capacity to identify new or emerging pathogens. NGS can be used to accurately identify and detect pathogens such as viruses, bacteria, fungi, and parasites in a variety of sample types, including clinical specimens, environmental samples, and vectors. The rapid and accurate identification of the pathogen responsible for an outbreak, the tracking of transmission patterns, and the monitoring of genomic alterations as an outbreak develops have all been made possible by NGS-based pathogen identification. Through the unbiased and thorough sequencing of microbial genomes made possible by NGS, new or genetically varied strains of bacteria can be found, in addition to well-known diseases. Additionally, NGS has proven crucial for the detection and tracking of resistance, the identification of zoonotic infections, and the understanding of the genetic diversity and evolution of pathogens in the surveillance and monitoring of infectious diseases.

However, there are also issues with using NGS for pathogen detection, such as the requirement for strong bioinformatics pipelines, the standardization of methods, and workflow optimization for various sample types. Data interpretation and analysis, which call on knowledge in bioinformatics and genetics, continue to be significant challenges. The application of NGS in recognizing clinical pathogens remains in the primary phase, and no mNGS procedures are approved by the Food and Drug Management Administration (Kim et al., 2016). Consequently, the current NGS procedures are personalized, and there is a long way to go to accomplish standardized detection (Kong et al., 2022). Moreover, the restricted capacity of NGS to detect low-frequency variations is one of its major drawbacks (Cohen et al., 2021). This restriction becomes important when there are several virus variants coexisting in quasispecies or when there are genetic differences in an organism known as heteroplasmy. Additionally, there are challenges with the clinical identification of pathogens. Currently, high diagnostic costs and a lack of genomics competence are the main barriers that prevent the adoption of NGS in clinics (Stoddard et al., 2014).

In conclusion, NGS has enormous potential in pathogen identification as it allows for more precise, quicker, and more thorough detection of infections. The application of NGS for identifying various pathogens will be further enhanced by ongoing developments in sequencing technologies, data analysis tools, and collaboration between researchers, clinicians, and public health agencies. These developments will also contribute to improve the diagnostics, surveillance, and control of infectious diseases.

## Author contributions

AN: Writing – original draft. YW: Writing – original draft. DW: Writing – original draft, Writing – review & editing. AS: Writing – original draft, Writing – review & editing. MA: Writing – original draft, Writing – review & editing. SX: Funding acquisition, Project administration, Writing – original draft, Writing – review & editing. YT: Funding acquisition, Project administration, Supervision, Writing – original draft, Writing – review & editing. All authors read and approved the final manuscript.
